# Weight changes after antiretroviral therapy initiation in CoRIS (Spain): a prospective multicentre cohort study

**DOI:** 10.1002/jia2.25732

**Published:** 2021-05-26

**Authors:** Javier Martínez‐Sanz, José‐Ramón Blanco, Alfonso Muriel, María Jesús Pérez‐Elías, Rafael Rubio‐Martín, Juan Berenguer, Joaquim Peraire, Enrique Bernal, Onofre Juan Martínez, Sergio Serrano‐Villar, Santiago Moreno, CoRIS

**Affiliations:** ^1^ Department of Infectious Diseases Hospital Universitario Ramón y Cajal IRYCIS Madrid Spain; ^2^ Department of Infectious Diseases Hospital San Pedro Centro de Investigación Biomédica de La Rioja (CIBIR) Logroño Spain; ^3^ Clinical Biostatistic Unit Departamento de Enfermería y Fisioterapia Hospital Universitario Ramón y Cajal Universidad de Alcalá IRYCIS CIBERESP Madrid Spain; ^4^ Hospital Universitario 12 de Octubre Madrid Spain; ^5^ Hospital General Universitario Gregorio Marañón Madrid Spain; ^6^ Hospital Universitari de Tarragona Joan XXIII IISPV Universitat Rovira i Virgili Tarragona Spain; ^7^ Hospital General Universitario Reina Sofía Murcia Spain; ^8^ Hospital General Universitario Santa Lucía Cartagena Spain

**Keywords:** HIV, antiretroviral agents, comorbidity, weight gain, obesity, body‐weight trajectory

## Abstract

**Introduction:**

Weight gain after starting antiretroviral therapy (ART) is a major problem that can increase morbidity. Our main objective was to evaluate the effects of initial ART on weight change in a large prospective cohort of HIV‐positive individuals.

**Methods:**

This was a prospective cohort study of 13,198 subjects included in the Spanish HIV Research Network (CoRIS) between January 2004 and November 2018. We included subjects who started triple ART and achieved HIV RNA suppression within 48 weeks. We fitted linear mixed models adjusted for potential confounders to compare longitudinal changes in weight. We used Cox proportional‐hazard models to compare treatment groups’ times to transition to a higher body mass index (BMI) category.

**Results:**

We analysed data from a total of 1631 individuals resulting in 14,965 persons/years and 14,085 observations. Individuals retained in the final multivariable model were representative of the overall cohort. NNRTI‐based first‐line ART was associated with a lower average weight gain compared to PI‐ (+0.7 kg per year, 95% CI 0.5 to 1.0, *p* < 0.001) and INSTI‐based (+0.9 kg per year, 95% CI 0.7 to 1.1, *p* < 0.001) regimens. Individuals starting ART with TAF+FTC had greater weight gain than those receiving TDF+FTC (+0.8 kg per year, 95% CI 0.3 to 1.4, *p* = 0.004). Women and black persons presented a greater weight gain than men and non‐black individuals. Differences in weight trajectories were driven mainly by changes during the first year of ART. The NNRTI group was less likely to transition from normal weight to overweight than the PI (aHR 1.48, 95% CI 1.18 to 1.85) and INSTI groups (aHR 1.30, 95% CI 1.03 to 1.64). PIs but not INSTIs were associated with a higher rate of overweight‐to‐obesity shift (aHR 2.17, 95% CI 1.27 to 3.72). No differences were found among INSTIs in the transition to a higher BMI category.

**Conclusions:**

INSTI‐ and PI‐based first‐line ARTs are associated with greater weight gain compared to NNRTI‐based ART. Within the NRTIs, TAF+FTC was most strongly associated with weight gain. This heterogeneous effect of ART on body weight could affect the long‐term risk of some non‐communicable diseases.

## 1 Introduction

Obesity is one of the greatest public health challenges of the 21st century [1, 2], and this epidemic does not spare people with HIV [3]. Since the introduction of highly effective treatments for HIV infection, there has been a shift from the weight loss that characterized HIV/AIDS in its early years to overweight or obesity, with prevalence similar to that observed in the general population [3].

In recent years, increasing data suggest that integrase strand transfer inhibitors (INSTIs) are associated with greater weight gain than other antiretrovirals [4, 5]. However, divergent results across different studies have sparked a debate over the effects of antiretroviral therapy (ART) on weight, which can conceptually be interpreted as a return‐to‐health or linked to a causal effect on weight changes, either gain or suppression [6]. Within the INSTI class, dolutegravir (DTG) and bictegravir (BIC) have been linked to a greater mean weight gain [5]. Nevertheless, some large cohort studies have found similar weight gain with the use of protease inhibitors (PI) and INSTIs [7, 8]. Moreover, recent findings indicate that there may be differences within the different nucleoside reverse transcriptase inhibitor (NRTI) backbones used. Among the NRTI combinations, tenofovir alafenamide (TAF)/emtricitabine (FTC) has been associated with the greatest weight gain [5, 9], although some of these studies should not be extrapolated to our setting, since they were performed in different populations [9]. Nevertheless, data on pre‐exposure prophylaxis (PrEP) with TAF+FTC demonstrate weight gain consistent with annual weight gain in the general population [10], as well as suppression of body fat accumulation with tenofovir disoproxil fumarate (TDF)/FTC compared to placebo, at least during the first 72 weeks from the start of treatment [11]. For this reason, the question has emerged as to whether the weight gain observed with TAF in switching studies is actually due to the discontinuation of TDF. The apparently suppressive effect of TDF+FTC on fat accumulation in the early stages of treatment requires further study.

The lack of consensus among the studies highlights the need to remain critical about whether the weight gain observed in patients on antiretroviral treatment is due to a generalized class effect of any specific group of drugs or whether lifestyle factors and demographics have a greater influence [12]. Given the important clinical consequences of weight gain, more data are needed to describe weight trajectories in large cohorts that collect data on people living with HIV from different socio‐demographic groups. Herein, we investigated the effects of initial ART on weight changes in a large, externally audited, prospective cohort of ART‐naïve HIV‐positive individuals.

## 2 Methods

### 2.1 Study design, participants, setting and eligibility

We analysed data from 13,198 subjects included in the Cohort of the Spanish HIV Research Network (CoRIS), which is a prospective multicentre cohort of HIV positive, treatment‐naïve adults recruited from 45 hospitals in Spain. All ART‐naïve patients who initiated follow‐up between 2 January 2004 and 30 November 2018 were included in a prospective clinical cohort study with continuous enrolment and standardized data collection [13]. We included all subjects who started triple ART, achieved HIV RNA suppression at 48 weeks, maintained suppressed viraemia during follow‐up under the same ART regimen, and had available weight measurements. We defined virological failure as two consecutive HIV RNA measurements >50 copies/mL. This study was approved by the Ethics Committee for Drug Research in La Rioja (Ref. CEImLAR P.I. 372) and by the Institutional Review Boards of the Carlos III Health Institute located in Madrid, Spain. All patients gave written informed consent upon entry into the cohort.

### 2.2 Statistical analysis

We performed a descriptive analysis of baseline characteristics using frequency distributions. We used linear mixed models with a first‐order autoregressive covariance structure to allow for correlation caused by repeated observations in the same subject. Interaction terms (time‐versus‐treatment group) were created to assess whether weight changes differed significantly over time between the treatment groups. To illustrate the predicted adjusted weight changes, we plotted the predicted weight gain means at 12‐month intervals, limiting the analyses to 36 months due to the shorter follow‐up in the INSTI group. Analyses were adjusted for age, sex, ethnicity, mode of transmission, calendar year, educational level, baseline HIV RNA, presence of AIDS, pre‐ART nadir CD4, backbone NRTI, baseline weight and height and the number of weight measurements. An additional model including the time from ART initiation to virological suppression as covariates did not yield different results. Subjects were censored at the last study visit or if they presented virological failure or changed their first‐line ART regimen. We performed a subgroup analysis including the subjects who received INSTI to assess the effect of the three drugs (DTG, elvitegravir [EVG] and raltegravir [RAL]) using the same methodology described above. Finally, we carried out a second subgroup analysis to evaluate the independent effect of the NRTI backbone, adjusted for the same covariates including the third drug used (non‐nucleoside reverse transcriptase inhibitor – NNRTI, PI or INSTI).

We then used Kaplan–Meier curves to calculate the time to and the rate of transition from normal weight to overweight, from overweight to obesity, and from class 1 obesity to class ≥2, and the cumulative probabilities. According to the WHO classification [14], we defined a normal weight as a BMI of 18.5 to 24.9 kg/m^2^, overweight as a BMI of 25 to 29.9 kg/m^2^ and obesity as a BMI of ≥30 kg/m^2^. Class ≥2 obesity was defined as a BMI ≥ 35 kg/m^2^. Changes from underweight to overweight or from normal weight to obesity were not considered because of the very low number of events (one and three respectively). Cox proportional‐hazard models, adjusted for the same covariates described above, were used to compare the times to the transition to a higher BMI category by treatment groups (NNRTI, PI and INSTI) and by INSTI (DTG, EVG and RAL). We evaluated the possible effects of the potential confounders on the transition to a higher BMI category with a stepwise backward variable selection process. The overall model was reapplied, including only the confounding factors that were retained in the model. Kaplan–Meier versus predicted survival plots with Breslow methods to handle tied failures was used to confirm that the proportional‐hazard assumptions were met. All statistical analyses were performed using Stata v. 15.1 (StataCorp LP, College Station, TX, USA).

## 3 Results

### 3.1 Characteristics of the study population

Of the 13,198 individuals included in the CoRIS cohort, 10,162 achieved undetectable plasma HIV RNA in the first 48 ± 6 weeks of ART initiation. Of these, 9672 started ART with a combination of two NRTI and either one NNRTI, one PI or one INSTI. A total of 1631 individuals contributing to 14,965 persons/years and 14,085 observations had available weight measurements, as well as data on all included covariates and were retained in the final multivariable model. The characteristics of the individuals not included due to missing data in the information needed for the statistical modelling strategy are shown in Table S1. The differences between groups in the analysed population were comparable to those found in the population with missing information with respect to the main variables included in the model, such as age, sex, mode of transmission, ethnicity, baseline weight and height and virological characteristics, such as nadir CD4 cell count, peak HIV‐1 RNA and time to virological suppression.

The median follow‐up was 47 months (IQR 28 to 75). Table 1 shows the population baseline characteristics. The study sample was representative of a middle‐aged population (median age 36 [IQR 30 to 44] years) with a higher representation of men (87%), a median baseline weight of 71 (IQR 64 to 79) kg, and a median baseline BMI of 23.7 (IQR 21.7 to 25.9) kg/m^2^. At baseline, 5% of participants were in the underweight category, 62% presented a normal weight, 26% were overweight and 7% were obese. Of these, 6% had class 1 obesity, and 1% had class ≥2 obesity. First‐line regimens included 637 subjects starting ART with two NRTI+NNRTI (39.1%), 342 subjects (21.0%) with two NRTI+PI and 652 subjects (40.0%) with two NRTI+INSTI. Among the INSTIs, 376 individuals (59%) received DTG, 159 individuals (25%) received EVG, and 107 individuals (17%) received RAL. Subjects in the PI group were more frequently injecting drug users, had a lower nadir CD4+ T‐cell count, had more frequently been diagnosed with AIDS, and had higher rates of virological failure during follow‐up.

**Table 1:** Population baseline characteristics according to the ART regimen

	2 NRTI + NNRTI (n = 637)	2 NRTI + PI (n = 342)	2 NRTI + INSTI (n = 652)	All (n = 1631)
Age, median (IQR)	36 (29‐44)	37 (30‐45)	36 (30‐45)	36 (30‐44)
Sex, n (%)				
Male	570 (89)	275 (80)	573 (88)	1418 (87)
Female	67 (11)	67 (20)	79 (12)	213 (13)
Mode of transmission, n (%)				
MSM	437 (69)	184 (54)	439 (67)	1060 (65)
Heterosexual	157 (25)	112 (33)	174 (27)	443 (27)
IDU	26 (4)	36 (11)	18 (3)	80 (5)
Other	3 (0.5)	0 (0)	2 (0.3)	5 (0.3)
Unknown	14 (2)	10 (3)	19 (3)	43 (3)
Origin, n (%)				
Spain	446 (70)	236 (69)	456 (70)	1138 (70)
Western Europe	11 (2)	15 (4)	18 (3)	44 (3)
Eastern Europe	20 (3)	4 (1)	10 (2)	34 (2)
Sub‐Saharan Africa	17 (3)	15 (4)	12 (2)	44 (3)
Northern Africa	10 (2)	3 (1)	7 (1)	20 (1)
Latin America	131 (21)	65 (19)	147 (23)	343 (21)
Other	2 (0.3)	4 (1)	1 (0.2)	7 (0.4)
Unknown	0 (0)	0 (0)	1 (0.2)	1 (0.1)
Ethnicity, n (%)				
Black	14 (2)	13 (4)	14 (2)	41 (3)
Non‐black	623 (98)	329 (96)	638 (98)	1590 (97)
Calendar period, n (%)				
2004‐2009	212 (33)	88 (26)	29 (4)	328 (20)
2010‐2014	375 (59)	181 (53)	187 (29)	743 (46)
2015‐2018	51 (8)	73 (21)	436 (67)	560 (34)
Education level, n (%)				
No studies	18 (3)	13 (4)	12 (2)	43 (3)
Primary	40 (6)	22 (5)	51 (8)	113 (7)
Secondary	124 (19)	94 (28)	92 (14)	310 (19)
High school	211 (33)	91 (27)	204 (31)	506 (31)
University	145 (23)	72 (21)	166 (25)	383 (23)
Other	11 (2)	13 (4)	16 (2)	40 (2)
Unknown	88 (14)	37 (11)	111 (17)	236 (14)
AIDS diagnosis, n (%)	47 (7)	52 (15)	64 (10)	163 (10)
Time to virological suppression (weeks), median (IQR)	24 (15‐38)	32 (19‐50)	14 (8‐30)	22 (12‐38)
Virological failure during follow‐up*, n (%)	49 (8)	36 (11)	21 (3)	106 (7)
Time to virological failure (weeks), median (IQR)	233 (179‐312)	195 (145‐301)	156 (143‐206)	204 (152‐273)
Duration on first‐line ART (months), median (IQR)	32 (13‐54)	24 (12‐44)	19 (11‐28)	23 (12‐41)
NRTI backbone, n (%)				
ABC+3TC	66 (10)	67 (20)	334 (51)	467 (29)
TDF+FTC	570 (89)	266 (78)	235 (36)	1071 (66)
TAF+FTC	1 (0.2)	9 (3)	83 (13)	93 (6)
Maximum HIV‐1 RNA (c/mL), median (IQR)	71500 (25767‐186200)	120041 (35962‐408848)	95690 (24919‐258276)	86334 (27400‐250000)
Number of HIV‐1 RNA quantifications, median (IQR)	67 (11‐23)	16 (10‐22)	8 (6‐12)	12 (8‐20)
Follow‐up (months), median (IQR)	64 (48‐91)	61 (37‐88)	28 (19‐40)	47 (28‐75)
Nadir CD4 cell count (cells/μL), median (IQR)	322 (229‐420)	236 (103‐346)	359 (211‐483)	316 (198‐435)
Weight (kg), median (IQR)	72 (65‐88)	69 (61‐78)	71 (64‐79)	71 (64‐79)
Height (cm), median (IQR)	174 (168‐178)	172 (167‐177)	173 (169‐178)	173 (168‐178)
BMI (kg/m2), median (IQR)	24 (22‐26)	24 (21‐26)	24 (22 to 26)	24 (22 to 26)
BMI category, n (%)				
Underweight	17 (3)	24 (7)	34 (5)	75 (5)
Normal weight	398 (62)	203 (59)	415 (64)	1016 (62)
Overweight	169 (27)	95 (28)	161 (25)	425 (26)
Obesity	53 (8)	20 (6)	42 (6)	115 (7)

3TC, lamivudine, ABC, abacavir; ART, antiretroviral therapy; BMI, body mass index; FTC, emtricitabine; IDU, injecting drug use; INSTI, integrase strand transfer inhibitor; MSM, men who have sex with men; NNRTI, non‐nucleoside reverse transcriptase inhibitor; NRTI, nucleoside reverse transcriptase inhibitor; PI, protease inhibitor; TAF, tenofovir alafenamide; TDF, tenofovir disoproxil fumarate.

* Subjects were censored on the date when virological failure was confirmed.

### 3.2 Weight gain by treatment groups

**Figure 1:**
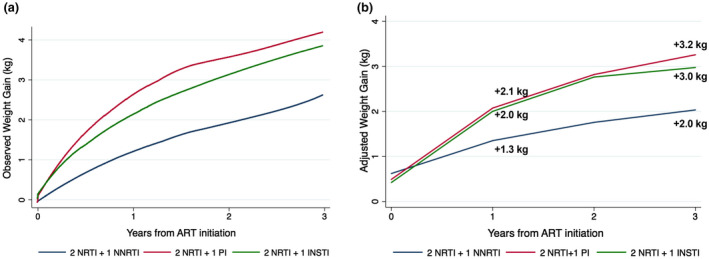
Effects of first‐line NNRTI‐, PI‐, and INSTI‐based ART on weight gain. The observed values are plotted in the left panel (a). The right panel (b) represents the predicted weight gain means at 12‐month intervals, adjusted for age, sex, ethnicity, mode of transmission, calendar year, educational level, baseline HIV RNA, presence of AIDS, pre‐ART nadir CD4, backbone NRTI, baseline weight and height, and number of weight measurements. Table S3 shows the predicted weight gain at six‐month intervals according to different treatment groups.

After adjustment for all covariates in the multilevel mixed model, factors associated with greater weight gain during follow‐up were as follows: lower weight (*p* < 0.001) and higher height (*p* = 0.005) at baseline, previous AIDS diagnosis (*p* < 0.001), lower CD4 nadir (*p* < 0.001), higher maximum HIV RNA (*p* = 0.001), and treatment with PI (*p* < 0.001) or INSTI (*p* < 0.001), compared to NNRTI. Table S2 shows the adjusted coefficients for all covariates included in the model. However, these should be interpreted with caution as they do not indicate the independent effect of each covariate on the weight trajectory. The observed weight trajectories and predicted weight gain by treatment regimen differed significantly among groups (*p* < 0.001) and are shown in Figure 1. In the global model, people who received PI and INSTI gained 0.7 kg (95% CI 0.5 to 1.0) and 0.9 kg (95% CI 0.7 to 1.1) more per year than those who received NNRTI (Table 2). Sub‐analyses adjusted for the use of either efavirenz (EFV) or rilpivirine (RPV) as NNRTI yielded similar results. Using PI as a reference, there were no statistically significant differences with INSTI (*p* = 0.112). After one year of ART, the mean adjusted weight gain was 1.3 (95% CI 1.0 to 1.7) kg for subjects receiving NNRTI, 2.1 (95% CI 1.6 to 2.5) kg for those with PI, and 2.0 (95% CI 1.6 to 2.4) kg for those receiving INSTI. After three years, the mean adjusted weight gain was 2.0 (95% CI 1.6 to 2.4) kg for those with NNRTI, 3.2 (95% CI 2.8 to 3.8) kg for those with PI, and 3.0 (95% CI 2.5 to 3.4) kg for those with INSTI (Table S3). Table S4 shows the predicted percentage of weight gain at various time points according to the different treatment groups.

**Table 2:** Comparison of weight gain trajectories (kg per year) between treatment groups using adjusted mixed‐effects models

Interaction term: year & ART group	Coefficient	95% CI	*p*
Overall model			
2NRTI + NNRTI	Ref.	–	–
2NRTI + 1PI	0.73	0.50, 0.97	<0.001
2NRTI + 1INSTI	0.94	0.73, 1.14	<0.001
*Subgroup analyses*			
**By sex**			
Female			
2NRTI + 1PI	1.53	0.88, 2.18	<0.001
2NRTI + 1INSTI	2.08	1.40, 2.75	<0.001
Male			
2NRTI + 1PI	0.60	0.35, 0.84	<0.001
2NRTI + 1INSTI	0.78	0.57, 0.99	<0.001
**By ethnicity**			
Black			
2NRTI + 1PI	2.06	0.62, 3.50	0.005
2NRTI + 1INSTI	2.20	0.67, 3.73	0.005
Non‐black			
2NRTI + 1PI	0.68	0.45, 0.93	<0.001
2NRTI + 1INSTI	0.90	0.69, 1.11	<0.001
**By treatment periods**			
First year of ART			
2NRTI + 1PI	1.81	1.30, 2.32	<0.001
2NRTI + 1INSTI	1.06	0.64, 1.48	<0.001
1 to 3 years of ART			
2NRTI + 1PI	0.17	−0.25, 0.58	0.437
2NRTI + 1INSTI	0.32	−0.04, 0.69	0.081

ART, antiretroviral therapy; CI, confidence interval; INSTI, integrase strand transfer inhibitor; NNRTI, non‐nucleoside reverse transcriptase inhibitor; NRTI, nucleoside reverse transcriptase inhibitor; PI, protease inhibitor; Ref, reference category.

Models adjusted for age, sex, ethnicity, mode of transmission, calendar year, educational level, baseline HIV RNA, presence of AIDS, pre‐ART nadir CD4, backbone NRTI, baseline weight and height, and number of weight measurements. An additional model including the time from ART initiation to virological suppression as covariates did not yield different results. A total of 1631 individuals were retained in the final multivariable model.

We then examined the different effects according to sex and ethnicity. We found greater weight gain among women and those of black ethnicity (Table 2). Moreover, sub‐analyses at different periods indicated that the differences found were driven by changes during the first year of ART without significant differences in the adjusted weight trajectories after the second year of ART. Hence, a stronger effect of PI and INSTI during the first year of ART determined greater weight gain after a similar follow‐up (Table 2).

Next, we asked whether the different INSTIs could lead to different changes in weight trajectories (Figure S1) and found no significant differences among INSTI drugs. After 36 months, the mean adjusted weight gain was 3.2 (95% CI 2.3 to 4.2) kg for those treated with DTG, 3.5 (95% CI 2.5 to 4.4) kg for those treated with EVG and 3.2 (95% CI 2.5 to 4.4) kg for those treated with RAL (Table S5). Finally, we assessed the effects of specific NRTI backbones. Patients starting ART with TAF+FTC had greater weight gain than those receiving TDF+FTC or ABC+3TC: 0.90 kg per year compared to ABC+3TC (95% CI 0.31 to 1.49, *p* = 0.003), and 0.83 kg per year compared to TDF+FTC (95% CI 0.26 to 1.40, *p* = 0.004) (Table S5, Figure S2).

**Figure 2:**
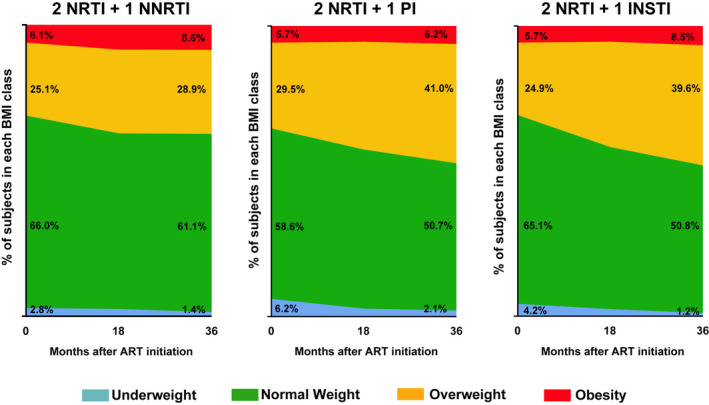
Changes in BMI class by treatment group. ART, antiretroviral therapy; BMI, body mass index; INSTI, integrase strand transfer inhibitor; NNRTI, non‐nucleoside reverse transcriptase inhibitor; NRTI, nucleoside reverse transcriptase inhibitor; PI, protease inhibitor.

**Figure 3:**
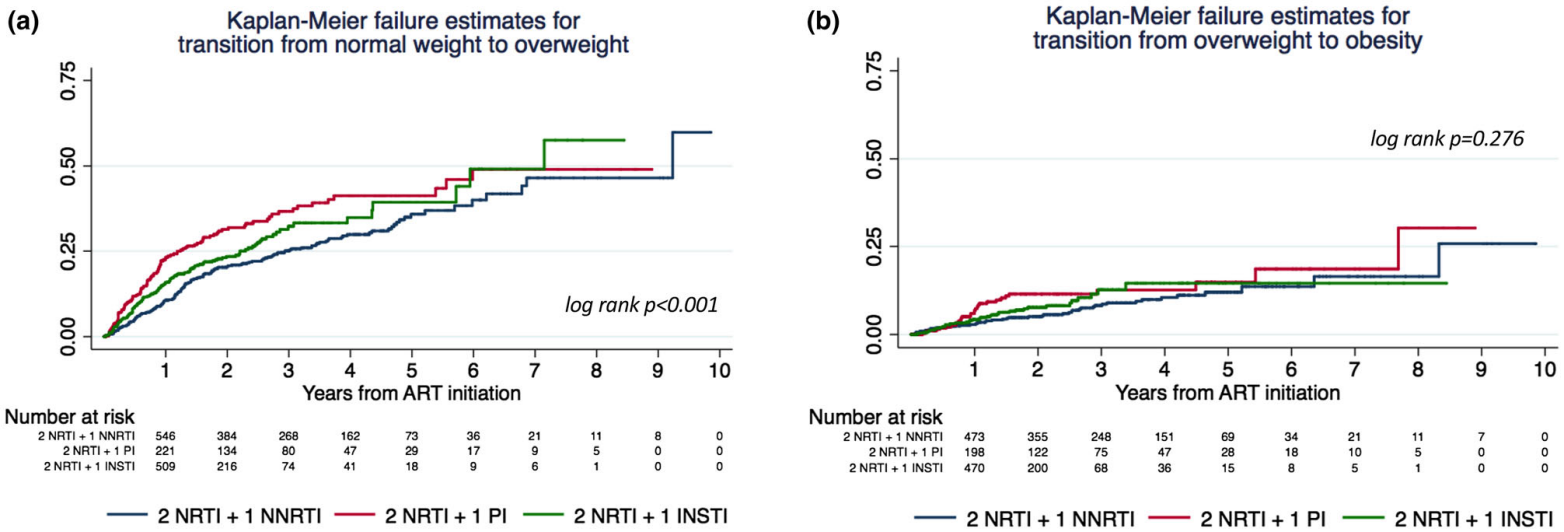
Kaplan–Meier survival plots of the estimated overall transition to a higher BMI category. ART, antiretroviral therapy; INSTI, integrase strand transfer inhibitor; NNRTI, non‐nucleoside reverse transcriptase inhibitor; NRTI, nucleoside reverse transcriptase inhibitor; PI, protease inhibitor.

We plotted the overall change in BMI class by treatment group, which is shown in Figure 2. At 36 months after ART initiation, patients on INSTI treatment presented a higher percentage of change to a higher BMI category, going from a prevalence of 25% of overweight people at the start of ART to 40% at 36 months, and from 5.7% obesity to 8.5% (*p* < 0.001). Figure 3 and Figure S3 show Kaplan–Meier survival plots of the estimated overall transition to a higher BMI category. The median (IQR) time to a transition from normal weight to overweight was 7.4 (2.8 to 14.2) months for INSTI, 8.4 (2.8 to 16.2) months for PI and 13.9 (5.9 to 24.3) months for NNRTI (log‐rank test *p* < 0.001). The median (IQR) time to a transition from overweight to obesity was 10.7 (4.4 to 16.7) months for INSTI, 11.3 (7.6 to 17.1) months for PI and 15.4 (3.7 to 34.7) months for NNRTI (log‐rank test *p* = 0.276). The incidence rates of transition to a higher BMI category by treatment group are reported in Table S6. The NNRTI group had a lower rate of transition from normal weight to overweight than the PI (aHR 1.48, 95% CI 1.18 to 1.85) and INSTI (aHR 1.30, 95% CI 1.03 to 1.64) groups (Table 3). No differences were found among INSTIs. The analysis according to the NRTI backbone showed a higher adjusted hazard ratio in patients receiving TAF+FTC, although not statistically significant (aHR 1.45, 95% CI 0.93 to 2.26, *p* = 0.099). PIs but not INSTIs were associated with a higher rate of overweight‐to‐obesity shift (aHR 2.17, 95% CI 1.27 to 3.72) (Table 3). Table S7 and S8 show the adjusted hazard ratios for all covariates included in the Cox proportional‐hazard models for the transition from normal weight to overweight and from overweight to obesity.

**Table 3:** Cox proportional‐hazard model for transition to a higher BMI category by treatment group

ART regimen	aHR	95% CI	*p*
Normal weight to overweight			
2NRTI + 1NNRTI	Ref.	‐	‐
2NRTI + 1PI	1.48	1.18, 1.85	0.001
2NRTI + 1INSTI	1.30	1.03, 1.64	0.032
Overweight to obesity			
2NRTI + 1NNRTI	Ref.	‐	‐
2NRTI + 1PI	2.17	1.27, 3.72	0.005
2NRTI + 1INSTI	1.19	0.68, 2.09	0.537

aHR, adjusted hazard ratio; ART, antiretroviral therapy; CI, confidence interval; INSTI, integrase strand transfer inhibitor; NNRTI, non‐nucleoside reverse transcriptase inhibitor; NRTI, nucleoside reverse transcriptase inhibitor; PI, protease inhibitor; Ref, reference category.

Models adjusted for age, sex, ethnicity, mode of transmission, calendar year, educational level, baseline HIV RNA, presence of AIDS, pre‐ART nadir CD4, backbone NRTI, baseline weight and height, and number of weight measurements. A total of 1631 individuals were retained in the final multivariable model.

## 4 DISCUSSION

In this observational study of a large prospective cohort of ART‐naïve, HIV‐positive individuals, we found that NNRTI‐based initial ART was associated with lower average weight gain compared to PI‐ and INSTI‐based regimens. However, no significant differences were found between the last two groups. Also, we observed that patients starting ART with TAF+FTC had greater weight gain than those receiving TDF+FTC or ABC+3TC. Women and those with black ethnicity experienced greater weight gain, and differences in weight trajectories were driven mainly by changes during the first year of ART.

Similar to our findings, prior studies have suggested that NNRTIs are associated with lower average weight gain [4, 5, 7]; however, weight gain trends vary greatly between different published works, and significant differences are not always found between INSTIs and PIs [7, 8]. In our cohort, we found no significant differences between PI‐ and INSTI‐based ART, showing similar weight gain with respect to NNRTI‐based ART, regardless of whether it was EFV or RPV. Regarding NNRTIs, RPV has been associated with more weight gain than EFV [5]. In the ADVANCE study, data found that CYP2B6 was identified as a relevant factor: Slow EFV metabolizers on EFV had minimal weight gain, whereas rapid metabolizers gained as much weight as those on DTG, arguing that it was the lack of EFV toxicity that accounted for the difference, rather than a direct DTG effect [15]. Regarding the comparison between INSTIs and PIs, the recent pooled analysis of eight ART‐naïve randomized clinical trials [5] revealed a greater average weight gain at two years among patients treated with INSTIs, with 3.2 kg versus 1.9 kg among those treated with NNRTIs and 1.7 kg with PIs. Conversely, a recent study in the NA‐ACCORD found no differences between INSTIs and PIs in the same period [4]. Data from observational cohorts revealed weight gain (+3 kg over 48 to 78 weeks) after switching to INSTI [16, 17]. In contrast, other switch studies showed that most of the population experienced small weight gains [18], or even no weight gain post‐switch [19]. In the NEAT‐022 trial [20], patients on PI‐based ART who switched to DTG experienced a weight gain of less than 1.25 kg over 96 weeks compared to those remaining in the PI group.

In our cohort, patients starting INSTI drugs showed a greater increase in the prevalence of overweight and obesity after three years of ART. Although the percentages are similar to, or even lower than, the prevalence of overweight and obesity in the non‐HIV population in Spain (37% and 17% respectively [21]), this increase is worrisome given the greater impact of obesity in people living with HIV [22], together with the infection itself.

Consistent with our findings, some recent publications have associated TAF with greater weight gain than TDF [5, 9, 23]. Nonetheless, there are major limitations that prevent the drawing of definitive conclusions in our setting, as most of these studies were carried out in Africa or have a retrospective design [9, 23]. Data from naïve patients in Cameroon (NAMSAL trial) [24] and South Africa (ADVANCE trial) [9] show a greater average 96‐week weight gain with DTG+TAF+FTC (+8 kg) compared to DTG+TDF+FTC (+5 kg) and EFV/TDF+FTC (+2 kg) (*p* < 0.001). Retrospective data from switch studies show consistent results with those observed in naïve patients, with BMI increases among patients who switched from TDF to TAF [25, 26]. Moreover, data from pooled clinical trials indicate that TAF use induces weight gain independent of concomitant INSTI use [5]. However, there is also evidence against the TAF+FTC‐associated weight gain. The DISCOVER trial showed a weight gain consistent with annual weight gain in the general population of the USA and Western Europe (0.5 to 1 kg/year). Furthermore, it showed results consistent with the weight‐suppressive and lipid‐suppressive effects of TDF [10]. Thus, some authors postulate that it is unlikely that one tenofovir prodrug (TDF) would cause weight loss and another (TAF) would cause weight gain, being more likely that TDF causes weight loss (a possible toxicity) and TAF does not.

The greater effect of drugs on weight gain among women and black people has been observed throughout the different cohorts and sub‐analyses of clinical trials [5, 18]. Different hypotheses have been postulated to explain these differences. Higher circulating leptin levels, subcutaneous adipose tissue metabolic rates, and more hypothalamic‐subcutaneous adipose tissue neuronal connections among women may, in part, explain these findings [27, 28]. Structural factors such as poverty, diet or physical activity, which might influence weight and are more prevalent among certain population groups, are usually overlooked in weight gain analyses, as most of these factors are not collected as adjustment variables in the cohorts. However, data from randomized clinical trials point in the same direction as the cohorts in terms of increased weight gain among black people.

It has been hypothesized that the greater weight gain observed with new drugs might be related to the fact that they are better‐tolerated and easier‐to‐take regimens, resulting in better ART adherence and (possibly) increased appetite and higher caloric intake [5]. To further address this point, we have considered the year of entry in the cohort as an adjustment variable. Additionally, we performed sensitivity analyses at three calendar periods, which did not reveal signals that the different year of entry in the cohort across groups was a significant source of bias.

Several limitations must be considered. The main limitation is the observational design, which we have tried to address by including diverse covariates; however, this could have resulted in residual confounders that interfered with the results. This fact has led to the exclusion of a large part of the cohort due to missing values, although the sample included is representative of the overall cohort. Similar to most cohorts, our analyses do not include bictegravir, as the analysis time preceded its introduction. In addition, the lower number of participants with TAF+FTC demands caution when interpreting the results. We could not adjust for exposure to other drugs associated with weight gain (e.g. psychotropic drugs) and behavioural factors such as alcohol or recreational drug use and physical activity. The low representation of women and black people might underestimate the effect of initial ART in populations with a higher prevalence of these groups.

## 5 Conclusions

This study provides new evidence reinforcing the notion that INSTI, PI and probably TAF are associated with weight gain. Patients in certain population groups are more affected, which should be considered in clinical practice when ART is initiated, especially in those patients with other added risk factors. Randomized clinical trials specifically designed to measure the effects of antiretrovirals on weight gain and mechanistic studies aimed at determining the cause of weight gain and/or suppression will be necessary to further advance our understanding of the health impact of ART beyond virological control.

## Competing interest

Outside the submitted work, J.M.‐S. reports non‐financial support from ViiV Healthcare, Gilead Sciences, and Jannsen Cilag. S. S.‐V. reports personal fees from ViiV Healthcare, Janssen Cilag, Gilead Sciences, and MSD as well as non‐financial support from ViiV Healthcare and Gilead Sciences and research grants from MSD and Gilead Sciences. S.M. reports personal fees and non‐financial support from ViiV Healthcare, Janssen, Gilead Sciences, and MSD, as well as grants from MSD, ViiV Healthcare, and Gilead Sciences. The authors report no potential conflicts of interest.

## Authors’ contributions

JR.B. and S.M. conceptualized the study; J.M.‐S., S.S.‐V. and S.M. analysed the data, generated the figures, and wrote the first draft of the manuscript; J.M.‐S, A.M., S.S.‐V. and S.M. contributed to writing and figures generation; J.M.‐S. and A.M. supervised the statistical analysis; J.M.‐S., J.R.B, A.M., M.J.P.‐E., R.R.‐M., J.B., J.P., E.B., O.J.M, S.S.‐V. and S.M. contributed to data mining. All authors revised and approved the final manuscript.

## Supporting information


**Table S1**. Baseline characteristics of population excluded because of missing information
**Table S2**. Adjusted multilevel mixed‐effects model for weight trajectories
**Table S3**. Predicted weight gain (95% confidence interval) at various time points according to different treatment groups.
**Table S4**. Predicted percentage of weight gain (95% confidence interval) at various time points according to different treatment groups.
**Table S5**. Comparison of weight gain trajectories (kg) by INSTI and NRTI backbone using adjusted mixed‐effects models.
**Table S6**. Incidence rates of transition to a higher BMI category.
**Table S7**. Cox proportional‐hazard model for transition from normal weigh to overweight
**Table S8**. Cox proportional‐hazard model for transition from overweight to obesity
**Figure S1**. Effects of different INSTI drugs on weight gain.
**Figure S2**. Effects of different NRTI backbones on weight gain.
**Figure S3**. Kaplan‐Meier survival plots of estimated transition from class 1 obesity to class ≥2 obesity.
